# Molecular Mechanisms of Aspartame-Induced Kidney Renal Papillary Cell Carcinoma Revealed by Network Toxicology and Molecular Docking Techniques

**DOI:** 10.3390/ijms27010077

**Published:** 2025-12-21

**Authors:** Chenjie Huang, Lulu Wei, Wenqi Yuan, Yaohong Lu, Gedi Zhang, Ziyou Yan

**Affiliations:** School of Clinical Medicine, Jiangxi University of Chinese Medicine, Nanchang 330004, China; huangchenjie@jxutcm.edu.cn (C.H.); 18702534935@163.com (L.W.); m15970290352@163.com (W.Y.); 13378268763@163.com (Y.L.)

**Keywords:** kidney renal papillary cell carcinoma, aspartame, network toxicology, molecular docking, SHAP analysis, survival analysis, molecular mechanism

## Abstract

Aspartame, a widely used artificial sweetener, has been linked to various cancers, including kidney renal papillary cell carcinoma (KIRP). However, the molecular mechanisms underlying this association remain unclear. This study employed network toxicology and molecular docking to investigate potential mechanisms of aspartame-induced KIRP. Differentially expressed genes from TCGA were intersected with aspartame targets and KIRP-related genes, yielding 61 common targets. GO and KEGG analyses revealed enrichment in extracellular matrix degradation, signaling pathways, and immune microenvironment regulation. Univariate Cox regression identified 23 prognostically significant genes, from which multifactorial Cox regression with stepwise selection determined 8 core genes (*APLNR*, *CYP2C19*, *EDNRA*, *KLK5*, *F2R*, *RAD51*, *AURKA*, and *TLR2*). A risk model was constructed and validated through VIF analysis, Schoenfeld residual testing, and internal validation using a training–validation split. SHAP analysis identified *EDNRA* as the primary driver gene. Survival analysis demonstrated that the model effectively stratified KIRP patients, with risk score and tumor stage serving as independent prognostic factors. Molecular docking confirmed stable binding between aspartame and core target proteins. These findings provide mechanistic insights into aspartame-induced KIRP pathogenesis and establish a foundation for future experimental validation.

## 1. Introduction

Aspartame is a synthetic non-nutritive sweetener that is approximately 200 times sweeter than sucrose. It is widely utilized in sugar-free beverages, nutritional supplements, chewing gum, and other low-sugar or sugar-free products [1]. Due to its minimal energy contribution and negligible effect on blood glucose levels, aspartame is often regarded as the preferred sugar substitute for individuals with diabetes and those seeking to lose weight [2]. Currently, aspartame is incorporated into more than 5000 food products globally, with a substantial population of chronically exposed consumers and a projected market size of $476.4 million by 2029 [3]. Although the U.S. Food and Drug Administration (FDA) has approved aspartame as a food additive and deems it safe for consumption within established limits, concerns regarding its safety and potential health risks have emerged as its prevalence in the global food supply chain increases. Notably, apprehensions about its potential carcinogenicity have been particularly emphasized [4]. In 2023, the International Agency for Research on Cancer (IARC) officially classified aspartame as a Group 2B carcinogen, indicating that it is “probably carcinogenic to humans” [5]. Following this classification, the FAO/WHO Expert Committee on Food Additives (JECFA) conducted a risk assessment and established an acceptable daily intake (ADI) for aspartame at 40 mg/kg body weight. Although some reports suggest a potential link between aspartame exposure and cancer development, the conclusions of existing studies primarily derive from animal experiments involving long-term or high-dose exposure, alongside limited epidemiological data [6]. These studies exhibit several limitations, including insufficient sample sizes, inconsistent findings, and the failure to exclude confounding factors, rendering the conclusion regarding carcinogenicity controversial. Therefore, it is essential to further investigate the toxicological properties of aspartame, particularly to clarify its association with cancer risk, which is crucial for ensuring food safety and protecting human health.

Renal cell carcinoma (RCC) is a prevalent malignant tumor of the urinary system, representing approximately 3–5% of all malignant tumors in adults [7]. The GLOBOCAN 2022 report indicates that RCC ranks 14th in incidence and 16th in mortality among all malignant tumors [8]. Kidney renal papillary cell carcinoma (KIRP) is the second most common subtype of RCC, comprising about 10–15% of all RCC cases [9]. KIRP primarily arises from the epithelium of the renal proximal tubule and is characterized by notable histological and molecular heterogeneity [10]. Although KIRP is primarily classified as a low-grade malignant tumor, advanced KIRP is susceptible to metastasis, resulting in a poor overall prognosis. Current treatment options for KIRP include surgery, VEGF inhibitors, immunotherapy, and mTOR inhibitors; however, the efficacy of these treatments in advanced KIRP remains limited [11]. Beyond genetic and molecular factors, environmental factors, such as exposure to pollutants, also influence the development of RCC [12]. Epidemiological research has demonstrated that exposure to environmental pollutants, including heavy metals and organic solvents, correlates with an increased risk of RCC. While many investigations have explored how environmental pollutants induce RCC, there is a paucity of studies addressing the potential carcinogenic effects of exogenous food additives, such as aspartame. Limited animal studies have indicated that high doses of aspartame may be linked to the pathogenesis of RCC; however, the precise molecular mechanisms remain unclear. Landrigan et al. [13] reported a significant increase in the incidence of lymphoma, leukemia, and RCC following chronic exposure of rodents to high doses of aspartame. The kidney, as a primary organ for metabolic clearance and excretion, is particularly vulnerable to toxic metabolites of ingested compounds. Aspartame metabolites, particularly formaldehyde, can induce oxidative stress and DNA damage, potentially affecting renal tubular epithelial cells from which KIRP originates [3]. However, systematic investigations specifically addressing the molecular mechanisms linking aspartame exposure to KIRP pathogenesis are lacking. Given that KIRP is the second most common RCC subtype yet remains relatively understudied compared to clear cell RCC, elucidating the potential mechanistic relationship between aspartame and KIRP is of significant scientific importance.

Traditional toxicological studies typically concentrate on a single target or pathway, which hinders a comprehensive understanding of the molecular mechanisms by which aspartame, a complex food additive, induces KIRP. In this study, we systematically elucidate the potential molecular targets, signaling pathways, and mechanisms associated with KIRP in response to aspartame exposure, utilizing network toxicology and molecular docking techniques. Network toxicology, an emerging research methodology, effectively addresses the limitations of traditional toxicology by integrating systematic information regarding compounds, targets, and diseases to identify key targets and pathways, thereby offering a novel perspective for elucidating the toxicity mechanisms of compounds [14]. Molecular docking predicts the interactions between compounds and target proteins identified through network toxicology, while also validating binding stability through binding energy calculations [15]. The integration of these two techniques establishes a closed loop of “target prediction–docking validation,” offering a distinct advantage in elucidating the mechanisms of toxic compounds. Building on this study, we developed a disease risk model by employing both unifactorial and multifactorial Cox regression analyses. We then assessed the model’s performance through SHAP interpretability analysis and survival analysis to investigate the molecular mechanisms of KIRP induced by aspartame from multiple perspectives. This approach aims to provide a scientific foundation for the identification of early risk markers, the stratification of high-risk populations, and the development of targeted therapies for KIRP. Additionally, it serves as a reference for the safety evaluation of aspartame as a food additive.

## 2. Results

### 2.1. Screening for Differentially Expressed Genes

**Figure 1:**
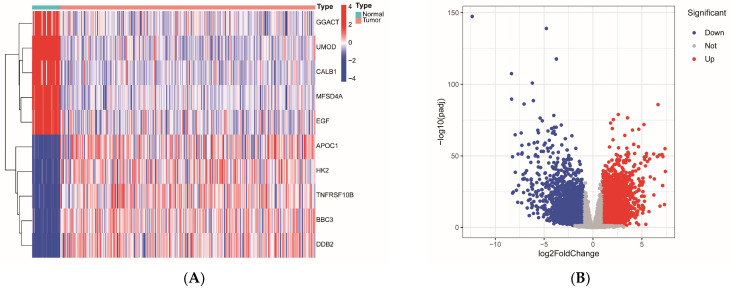
Visualization of differentially expressed genes in KIRP: (**A**) Heatmap displaying expression patterns of the top 5 most significantly upregulated and top 5 downregulated genes across 291 tumor samples and 32 normal samples. Expression values were z-score normalized, with red indicating high expression and blue indicating low expression. Hierarchical clustering was applied to both rows (genes) and columns (samples). The complete heatmap of the top 50 genes in each category is provided in Appendix A (Figure A1). (**B**) Volcano plot illustrating the distribution of 5022 differentially expressed genes. The *x*-axis represents log2 fold change (tumor vs. normal), and the *y*-axis represents −log10(*p*-value). Genes were classified according to the following criteria: upregulated (red): log2FC > 1 and *p* < 0.05; downregulated (blue): log2FC < −1 and *p* < 0.05; non-significant (gray): |log2FC| ≤ 1 or *p* ≥ 0.05. The non-overlap between red and blue points reflects the mutually exclusive nature of positive and negative fold-change values.

The RNA-seq data for KIRP, sourced from the TCGA database, comprised 291 tumor samples and 32 normal samples. A total of 5022 differentially expressed genes were identified, including 2193 downregulated and 2829 upregulated genes. A heatmap was constructed to display the expression patterns of representative differentially expressed genes across individual samples, preserving sample-level resolution and enabling identification of expression clusters that would be obscured in aggregate distribution plots. The top 5 upregulated and top 5 downregulated genes are shown in Figure 1A (complete heatmap of top 50 genes in each category provided in Appendix A). A volcano plot was generated to illustrate the overall distribution of all differentially expressed genes (Figure 1B), where genes with log2(fold change) > 1 and *p* < 0.05 were classified as upregulated (red), genes with log2(fold change) < −1 and *p* < 0.05 as downregulated (blue), and remaining genes as non-significant (gray).

### 2.2. Analysis of Intersecting Target Genes of Aspartame and KIRP

**Figure 2:**
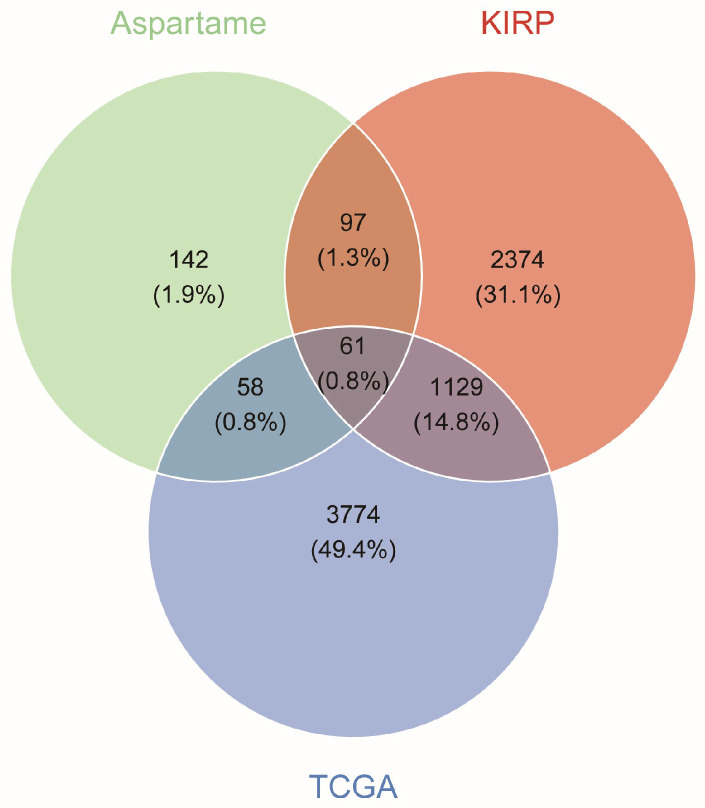
Target identification of aspartame in KIRP. Venn diagram depicting the intersection of differentially expressed genes from TCGA (blue, *n* = 5022), potential target genes of aspartame (green, *n* = 358), and KIRP-associated targets (red, *n* = 3661). The three-way intersection yielded 61 common target genes. All percentages shown represent the proportion of genes in each region relative to the total union of all three datasets (*n* = 7635).

Using the complementary databases ChEMBL, SwissTargetPrediction, and SEA, systematic predictions were conducted with “Aspartame” as the keyword. After merging and deduplicating the results, 358 potential target genes for aspartame were collected. Additionally, a search of the GeneCards, OMIM, and CTD databases with the keyword “Kidney renal papillary cell carcinoma” yielded 3661 disease-related targets for KIRP, also following a merging and deduplication process. The 5022 differentially expressed genes, aspartame target genes, and KIRP-related targets obtained from the TCGA database were subsequently combined and intersected, leading to the identification of 61 intersected target genes through which aspartame may influence the pathogenesis of KIRP (Figure 2). Detailed information on each gene set in the Venn diagram are presented in Supplementary Materials Table S1.

### 2.3. GO and KEGG Pathway Enrichment Analysis of Intersecting Target Genes

**Figure 3:**
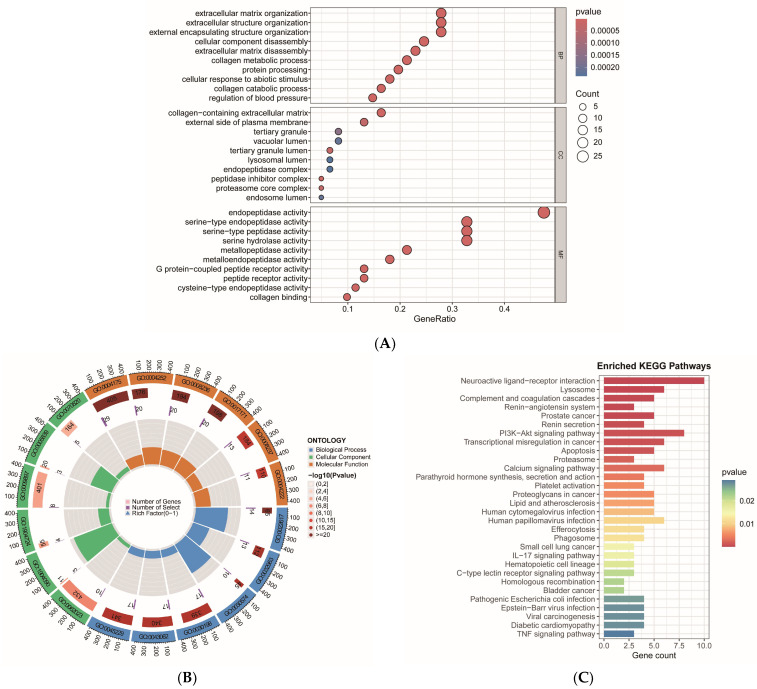
GO and KEGG enrichment analyses were conducted on intersecting target genes: (**A**) The GO bubble diagram illustrates the annotation of these intersecting genes across BP, CC, and MF. The *x*-axis represents the gene ratio, which is calculated as the number of genes from the target gene set belonging to a specific GO term divided by the total number of genes in that GO term. The *y*-axis displays the names of the GO functional entries. The color gradient indicates the corrected *p*-value, with a deeper red signifying greater significance. The size of the bubbles corresponds to the number of enriched genes, with larger bubbles indicating a higher gene count. (**B**) The GO circle diagram provides a detailed view of gene enrichment across various GO entries. The outermost circle scale represents the total number of genes, expressed as a power of 10. The first circle denotes the pathway number and classification. The length of the rectangles in the second circle reflects the number of genes associated with each term, with darker colors indicating higher enrichment significance. The length of the rectangles in the third circle represents the number of genes overlapping with the GO entry in the input gene set. The fourth circle illustrates the ratio of the number of genes in the third circle to the corresponding number of genes in the second circle. (**C**) The KEGG histogram illustrates the enriched pathways of the intersecting genes. The *x*-axis denotes the number of genes within each pathway, while the *y*-axis lists the names of the pathways. The color gradient of the bars indicates the corrected *p*-value, with a deeper red signifying greater significance.

Functional characterization of the intersecting targets was conducted through GO and KEGG enrichment analyses (Figure 3A–C) to elucidate the molecular mechanisms by which aspartame influences the development of KIRP. The results of the GO analysis indicated significant enrichment in biological processes (BP), including extracellular matrix degradation, collagen metabolism and catabolism, and extracellular structural organization. These findings suggest that aspartame may facilitate KIRP invasion and metastasis by remodeling the tumor microenvironment. In cellular components (CC), enrichment was observed in the collagen-rich extracellular matrix, proteasome core complex, endosomes, and lysosomal compartments, implying that aspartame may impact extracellular matrix remodeling and protein degradation, thereby affecting tumor cell growth and metabolic reprogramming. In molecular functions (MF), enrichment in protease activity, G protein-coupled receptor activity, and collagen-binding function was noted, highlighting the roles of protein hydrolysis, signal transduction, and extracellular matrix interactions in KIRP. KEGG pathway analyses revealed enrichment in neuroactive ligand-receptor interaction pathways, classical signaling pathways (e.g., PI3K-Akt, TNF, IL-17), tumor-associated pathways (e.g., prostate cancer, small cell lung cancer, bladder cancer), and metabolic and structural pathways (e.g., lipid and atherosclerosis, proteoglycans, proteasome, and calcium signaling pathways), as well as pathways related to viral infections (e.g., Epstein–Barr virus, human papillomavirus, human cytomegalovirus), immune responses (e.g., phagosome, lysosome), and cell death (e.g., apoptosis). These pathways encompass biological processes such as tumor cell proliferation, apoptosis, immune evasion, metabolic reprogramming, drug resistance mechanisms, and viral infection mechanisms. In summary, aspartame may facilitate the remodeling of the KIRP tumor microenvironment by regulating extracellular matrix degradation, signaling pathway transduction, protein metabolism, and the immune microenvironment, thereby accelerating disease progression.

### 2.4. Construction and Analysis of PPI Networks

**Figure 4:**
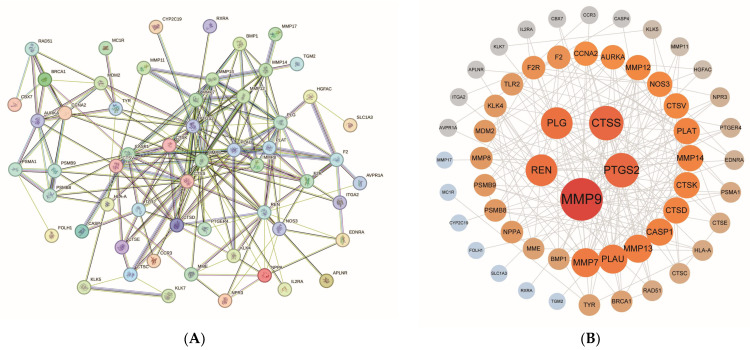
PPI network construction and visualization analysis: (**A**) The PPI network illustrates a graph constructed from 61 intersecting target genes, where each node represents a protein and the edges signify inter-protein interactions. Higher node densities indicate stronger functional associations among proteins. (**B**) The visualization analysis of the PPI network reveals the strength of protein interactions. Node color, ranging from gray to red, and node size, varying from small to large, correspond to degree values. Specifically, red and larger nodes indicate genes with higher degree values, whereas gray and smaller nodes represent genes with lower degree values. This indicates that as the degree value increases, this class of proteins interacts more robustly with other targets.

The 61 intersecting target genes identified from the screening were imported into the STRING database to construct a PPI network. This network comprised 57 protein nodes and 203 edges. The PPI network file was subsequently imported into Cytoscape v3.10.3, where it was further screened and sorted based on degree value. The results were visualized (Figure 4A,B), with darker colors and larger sizes indicating stronger interactions with other proteins.

### 2.5. Cox Regression Analysis and Risk Modeling

**Figure 5:**
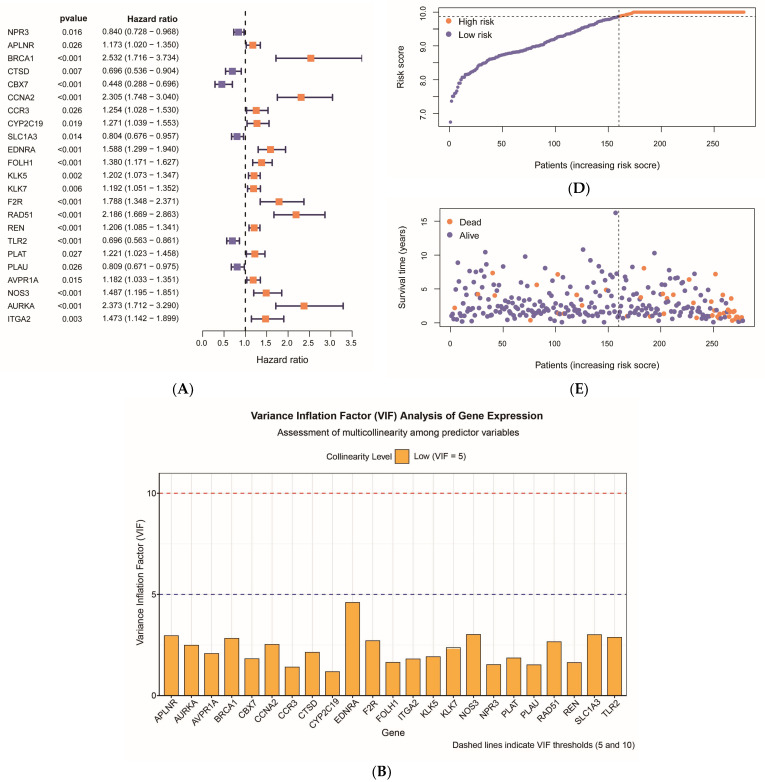

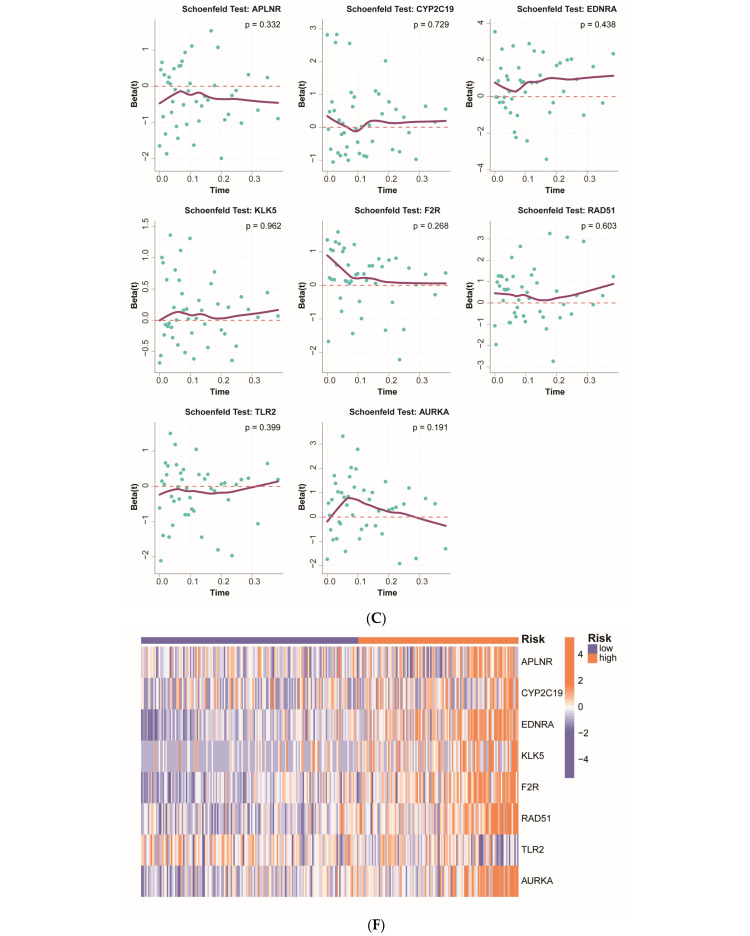
Construction and assessment of the risk model: (**A**) The forest plot illustrates 23 genes significantly associated with prognosis (*p* < 0.05), initially identified through unifactorial Cox regression analysis. This plot includes the hazard ratio (HR), 95% confidence interval, and *p*-value for each gene. The orange and purple horizontal lines represent the confidence intervals of the HR, with orange indicating HR > 1 (risk factor) and purple indicating HR < 1 (protective factor). The length of each horizontal line reflects the range of the confidence intervals, while squares denote point estimates of HR. (**B**) Variance inflation factor (VIF) analysis for the 23 prognostically significant genes. The bar chart displays VIF values for each gene in alphabetical order, with dashed lines indicating thresholds for low collinearity (VIF = 5, blue) and severe collinearity (VIF = 10, red). All genes exhibited VIF values below 5 (range: 1.18–4.60), confirming the absence of significant multicollinearity. (**C**) Schoenfeld residual plots for the 8 core genes in the multifactorial Cox regression model. Each panel displays the scaled Schoenfeld residuals plotted against time, with green dots representing the estimated regression coefficients at each time point. The solid line representing a smoothed spline fit and the dashed horizontal line at zero indicating perfect proportional hazards. *p*-values for each gene are shown, and the global test (*p* = 0.423) confirmed no significant violation of the proportional hazards assumption. (**D**) Risk score curves depict the distribution characteristics of KIRP patients sorted by increasing risk score. In this representation, purple dots indicate low-risk patients, orange dots denote high-risk patients, and the dashed line marks the boundary between the low and high-risk groups. (**E**) Survival status distribution plots illustrate the survival characteristics of KIRP patients, also sorted by increasing risk score. Here, purple dots represent surviving patients, orange dots indicate deceased patients, and the dotted line delineates the high and low-risk groups. (**F**) The core gene expression heatmap displays the expression distribution of eight core genes in KIRP patients, further refined through multifactorial Cox regression analysis. In this heatmap, purple indicates low expression, white represents moderate expression, and orange signifies high expression.

The gene expression data and survival information, including survival time and status, for the intersecting gene list and KIRP samples from the TCGA database were integrated. This process yielded a total of 278 tumor group samples for subsequent analysis after retaining the relevant information. An unifactorial Cox regression analysis was then conducted on 61 intersecting target genes using the R programming language, resulting in the identification of 23 genes significantly associated with patient prognosis (*p* < 0.05). Among these, 17 were classified as high-risk genes and 6 as low-risk genes (Figure 5A). Prior to multifactorial Cox regression, multicollinearity diagnostics were performed on the 23 prognostically significant genes. The variance inflation factor (VIF) analysis revealed that all candidate genes exhibited VIF values below 5 (range: 1.18–4.60; mean VIF = 2.28), with *EDNRA* showing the highest VIF (4.60) and *CYP2C19* showing the lowest (1.18) (Figure 5B). These results confirmed the absence of significant multicollinearity, satisfying the modeling assumptions for subsequent multifactorial Cox regression analysis. Subsequently, these significantly related genes were included in a multifactorial Cox regression analysis and further refined using the stepwise regression method based on the AIC criterion. Ultimately, 8 independent prognostic genes and their corresponding regression coefficients were identified (Table 1). Among the 8 core genes, *APLNR* (Coeff = −0.285) and *TLR2* (Coeff = −0.188) exhibited negative coefficients, indicating their protective roles in KIRP prognosis, whereas the remaining six genes with positive coefficients represent risk factors. The proportional hazards assumption was evaluated for the 8-gene Cox regression model using Schoenfeld residual analysis. The global test demonstrated no significant violation of the assumption (χ^2^ = 8.106, df = 8, *p* = 0.423). Individual gene tests also confirmed compliance: *APLNR* (*p* = 0.332), *CYP2C19* (*p* = 0.729), *EDNRA* (*p* = 0.438), *KLK5* (*p* = 0.962), *F2R* (*p* = 0.268), *RAD51* (*p* = 0.603), *TLR2* (*p* = 0.399), and *AURKA* (*p* = 0.191) (Figure 5C). These findings validated the appropriateness of the Cox proportional hazards model for this dataset. A risk model was constructed based on these core genes, and the risk score for each sample was calculated. The samples were then categorized into a high-risk group (118 cases) and a low-risk group (160 cases) based on the optimal risk score threshold of 10.256, which was utilized for subsequent risk model assessment and survival analysis. After arranging the samples in ascending order based on the risk score, the plotted risk score curves revealed distinct boundaries between high- and low-risk groups as the risk score increased. The risk scores for samples in the high-risk group were significantly greater than those in the low-risk group (Figure 5D). This finding demonstrates that the model effectively differentiates between samples across varying risk tiers. The survival status distribution graphs indicated that as the risk score increased, fatal events predominantly occurred in the high-risk group, where the number of deaths was significantly greater than in the low-risk group. Consequently, the survival rate of the low-risk group was higher (Figure 5E), thereby validating the efficacy of the risk model in prognostic stratification. Furthermore, the expression heatmap of the core genes within the risk model revealed that, with rising risk scores, the expression levels of *APLNR*, *CYP2C19*, *EDNRA*, *KLK5*, *F2R*, *RAD51*, and *AURKA* tended to be upregulated, while *TLR2* expression was gradually downregulated (Figure 5F). This finding demonstrated a significant difference in core gene expression levels between the high and low-risk groups, suggesting that the expression patterns of these genes are closely associated with tumor biological progression and patient prognosis. This further substantiates the rationale and potential applicability of the risk model in both biological and clinical contexts.

### 2.6. Explanatory Analysis of SHAP-Based Risk Models

**Figure 6:**
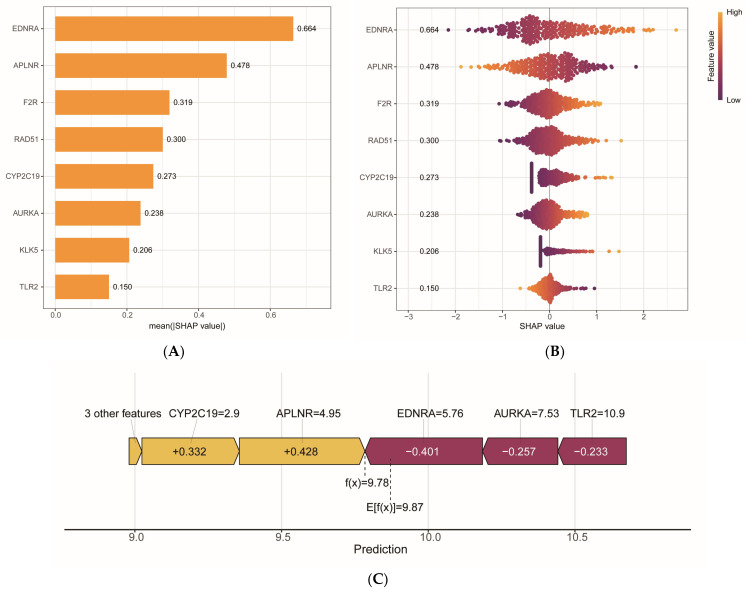
Interpretation of the SHAP-based risk model: (**A**) The SHAP feature importance histogram illustrates the top eight genes ranked by the average absolute SHAP value, where longer bars indicate a greater average contribution of the gene to the overall predicted outcomes of the model. (**B**) The SHAP summary plot depicts the distribution of SHAP values for each gene across all samples. The *x*-axis represents the SHAP value, while the *y*-axis denotes the gene name. Each point corresponds to a sample, and the color indicates the expression level of the respective gene in that sample (Feature value); purple signifies low expression, and orange indicates high expression. The width of the graph reflects the density of samples within a specific SHAP value interval. (**C**) The SHAP force plot illustrates the contributions of genes to the prediction results. A positive SHAP value signifies that the gene influences the model output towards a higher risk probability, with orange representing an increase in risk. Conversely, a negative SHAP value indicates that the gene drives the model output towards a lower risk probability, with purple representing a decrease in risk.

To elucidate the contribution of each core gene to the risk prediction model, this study employed the SHAP algorithm to analyze the systematic interpretability of the model. The SHAP feature importance histogram (Figure 6A) indicated that *EDNRA* (SHAP value = 0.664) had the greatest impact on model prediction performance, followed by *APLNR*, *F2R*, *RAD51*, *CYP2C19*, *AURKA*, *KLK5*, and *TLR2*. These findings suggest that these genes are critical variables for KIRP risk prediction. Furthermore, the SHAP summary plot (Figure 6B) illustrated the relationship between the expression levels of each gene and the model’s predicted output. A positive SHAP value indicates that increased gene expression enhances high-risk predictions, while a negative value suggests a preference for low risk. The analysis revealed that elevated expression of *EDNRA*, *F2R*, *RAD51*, *CYP2C19*, *AURKA*, and *KLK5* was positively correlated with an increased risk of KIRP, whereas the expression of *APLNR* and *TLR2* was associated with a reduced risk. Additionally, the SHAP force plot (Figure 6C) for a single representative sample demonstrated the contribution of each gene’s expression to individual risk prediction. The risk prediction score for this sample was f(x) = 9.78, which is lower than the overall mean value E[f(x)] = 9.87. The contributions of *CYP2C19* and *APLNR* were +0.332 and +0.428, respectively, which elevated the prediction score above the mean. Conversely, *EDNRA*, *AURKA*, and *TLR2* contributed −0.401, −0.257, and −0.233, respectively, resulting in a score below the mean. Notably, although *EDNRA* is generally considered a high-risk gene, the SHAP values for this sample indicated a mitigating effect on risk prediction due to interactions with other features. This observation underscores the SHAP method’s capacity to provide local explanations for individual samples and highlights the model’s nonlinear responses to complex feature combinations, consistent with theoretical expectations of SHAP. In summary, the SHAP analysis not only quantified the importance of features and their association with gene expression in risk prediction within the multigene model but also illuminated the individual heterogeneity of model decisions. Furthermore, it emphasized the pivotal role of *EDNRA* in regulating KIRP risk as a key driver of the model’s predictive capability.

### 2.7. Survival Analysis of Risk Models

**Figure 7:**
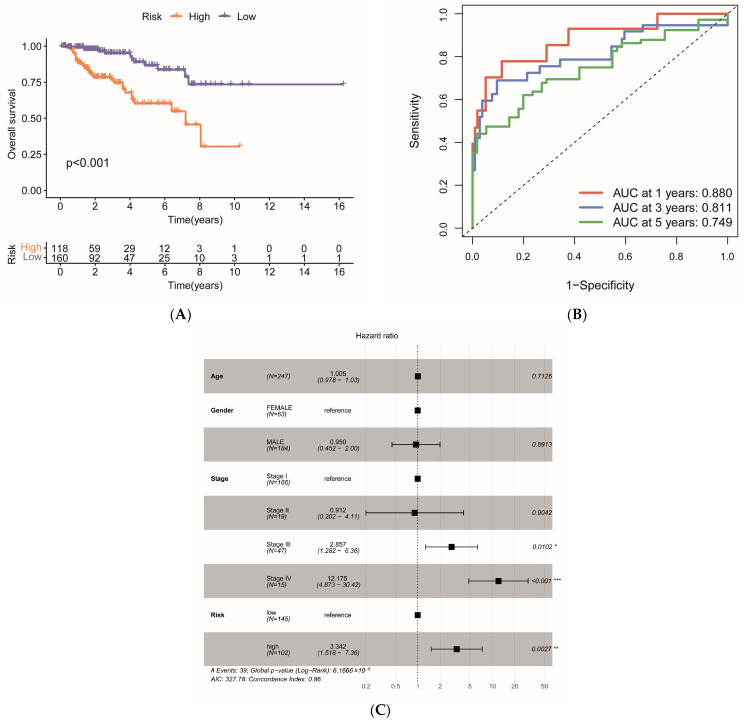
Survival analysis of the risk model: (**A**) K-M curves illustrate the survival probabilities of each group over time after categorizing KIRP patients into high- and low-risk groups based on the risk score. The *x*-axis represents different time points, while the *y*-axis indicates the cumulative survival rate. The *p*-value, calculated using the Log-rank test, assesses whether the survival differences between the high- and low-risk groups are statistically significant. (**B**) ROC curves depict the accuracy of the risk scoring model in predicting 1-, 3-, and 5-year survival. In each ROC curve, the *x*-axis denotes the false-positive rate, the *y*-axis represents sensitivity, with the dashed line indicating the baseline performance of a random model (AUC = 0.5). The AUC value reflects the model’s predictive performance at various time points, with values closer to 1 indicating superior predictive capability. (**C**) Forest plots present the results of multifactorial Cox regression analyses based on age, gender, staging, and risk group. The dashed line indicates the point where the hazard ratio is 1, which is used to assess whether the confidence interval is statistically significant. The # symbol represents the total number of events that occurred in the cohort. The * symbols indicate statistical significance, with * denoting *p* < 0.05, ** denoting *p* < 0.01, and *** denoting *p* < 0.001.

The K m curves demonstrated a statistically significant difference in overall survival time between the two sample groups (*p* < 0.001), with the high-risk group exhibiting shorter survival compared to the low-risk group, indicating a poorer prognosis for the high-risk group (Figure 7A). The AUC values from the ROC curves for predicting 1-, 3-, and 5-year overall survival in KIRP patients were 0.880, 0.811, and 0.749, respectively (Figure 7B). These results suggest that the constructed 8-gene model possesses strong predictive capability at 1, 3, and 5 years, particularly demonstrating high accuracy in predicting 1-year survival, which holds potential clinical application value, although it requires supplementation with additional biomarkers. The TCGA risk score was incorporated into the multifactorial Cox regression analysis alongside clinicopathologic characteristics, including age, gender, and stage. The findings revealed that the risk score (HR = 3.342, 95% CI: 1.518–7.359, *p* = 0.003) serves as an independent prognostic factor influencing survival outcomes in KIRP, maintaining robust predictive value. Furthermore, stage emerged as a significant independent risk factor, particularly for stage III (HR = 2.857, 95% CI: 1.282–6.364, *p* = 0.010) and stage IV (HR = 12.175, 95% CI: 4.873–30.420, *p* < 0.001), suggesting that patients with advanced KIRP experience a poorer prognosis, with a marked increase in mortality risk as the disease progresses. The risk ratios and confidence intervals of specific variables are detailed in Figure 7C.

### 2.8. Internal Validation of the Risk Model

To assess the generalizability of the 8-gene risk model, internal validation was performed by randomly splitting the 278 KIRP samples into a training set (*n* = 194, 70%) and a validation set (*n* = 84, 30%). Risk scores were calculated using the regression coefficients derived from the original multifactorial Cox regression model, and patients were stratified into high- and low-risk groups based on the median risk score of the training set.

**Figure 8:**
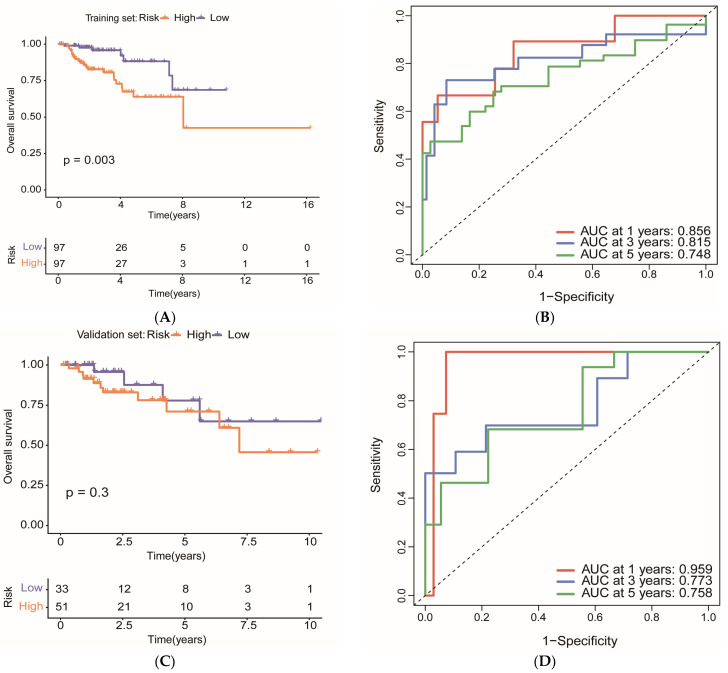
Internal validation of the 8-gene risk model: (**A**) Kaplan–Meier survival curves for the training set (*n* = 194). Patients were stratified into high-risk (*n* = 97, orange) and low-risk (*n* = 97, purple) groups based on the median risk score. The log-rank test demonstrated a significant difference in overall survival between the two groups (*p* = 0.003). (**B**) Time-dependent ROC curves for the training set evaluating the predictive accuracy of the risk model at 1, 3, and 5 years. AUC values were 0.856, 0.815, and 0.748, respectively, with the dashed line indicating the baseline performance of a random model (AUC = 0.5). (**C**) Kaplan–Meier survival curves for the validation set (*n* = 84). Although statistical significance was not reached (*p* = 0.3) due to limited sample size, the survival trend was consistent with the training set, with the high-risk group (*n* = 51) showing lower survival probability than the low-risk group (*n* = 33). (**D**) Time-dependent ROC curves for the validation set. AUC values were 0.959, 0.773, and 0.758 for 1-, 3-, and 5-year survival, respectively, and the dashed line indicating the baseline performance of a random model (AUC = 0.5), thus demonstrating excellent predictive performance.

In the training set, Kaplan–Meier analysis demonstrated a significant difference in overall survival between high-risk (*n* = 97) and low-risk (*n* = 97) groups (*p* = 0.003), confirming the prognostic stratification capability of the model (Figure 8A). Time-dependent ROC analysis showed that the model achieved AUC values of 0.856, 0.815, and 0.748 for predicting 1-, 3-, and 5-year survival, respectively (Figure 8B).

In the validation set, although the log-rank test did not reach statistical significance (*p* = 0.3) due to the limited sample size (*n* = 84), the survival curves showed a consistent trend with the high-risk group (*n* = 51) exhibiting lower survival probability compared to the low-risk group (*n* = 33) (Figure 8C). Importantly, the ROC analysis in the validation set demonstrated excellent predictive performance, with AUC values of 0.959, 0.773, and 0.758 for 1-, 3-, and 5-year survival, respectively (Figure 8D). The remarkably high 1-year AUC (0.959) in the validation set strongly supports the robustness and generalizability of the 8-gene risk prediction model.

### 2.9. Molecular Docking Results

**Figure 9:**
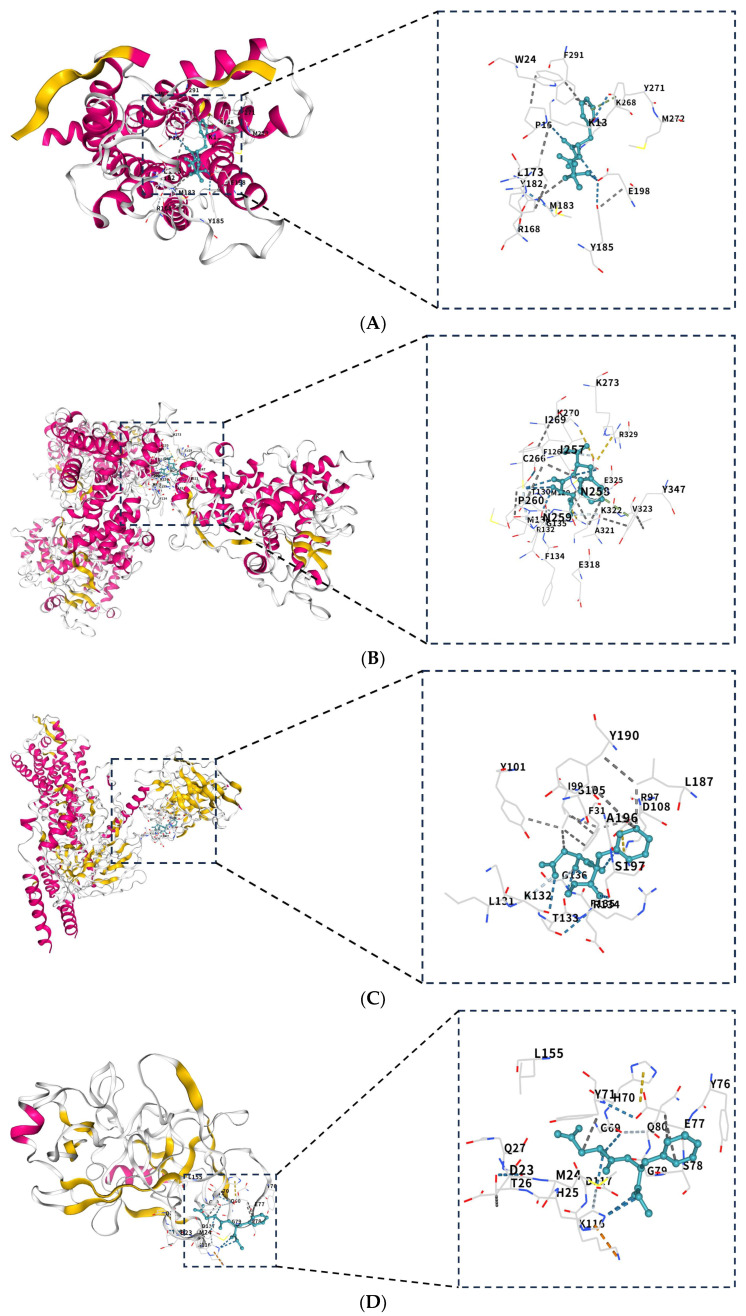

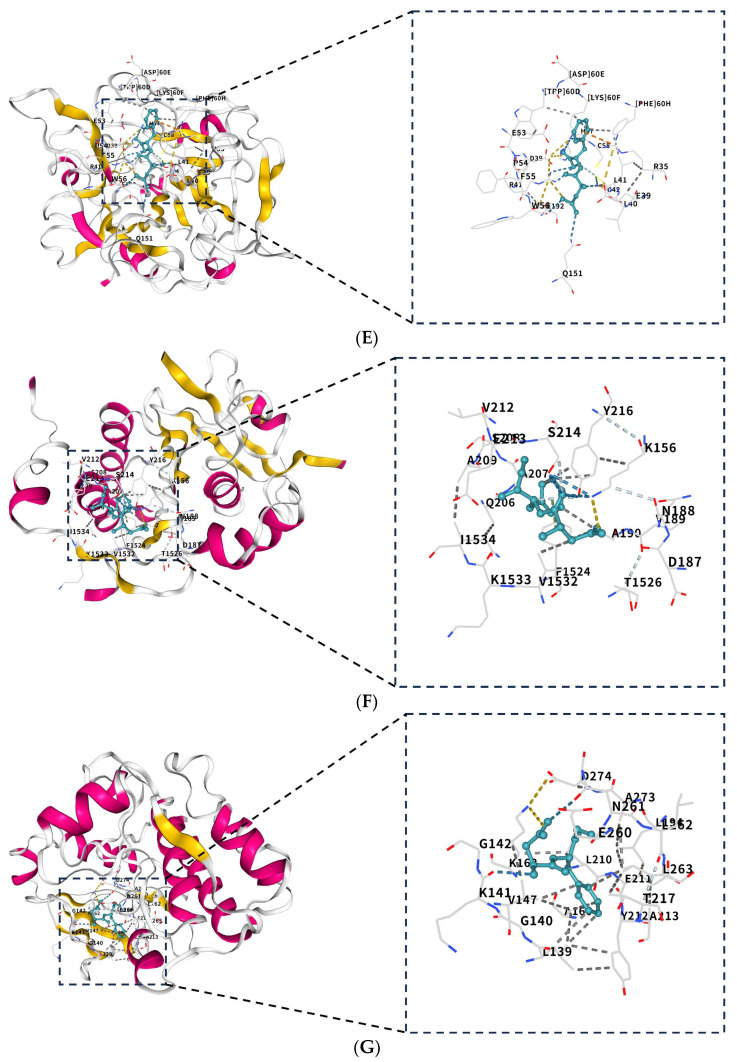

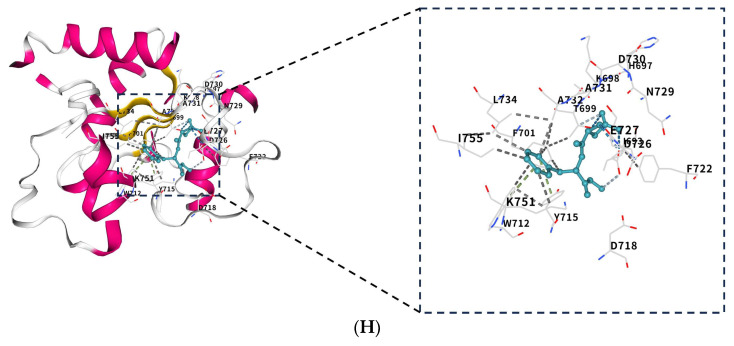
Molecular docking results of aspartame-core genes: (**A**) aspartame–APLNR; (**B**) aspartame–CYP2C19; (**C**) aspartame–EDNRA; (**D**) aspartame–KLK5; (**E**) aspartame–F2R; (**F**) aspartame–RAD51; (**G**) aspartame–AURKA; (**H**) aspartame–TLR2. The molecular docking diagram illustrates the overall complex structure and highlights key regions. Cyan represents the ligand, yellow highlights the active site, white and gray denote other parts of the protein, and pink represents secondary structural elements (e.g., α-helices). The dashed lines connect the amino acid residues with the ligand, indicating interactions between these residues and the ligand (e.g., hydrogen bonds, hydrophobic interactions), and the letter labels denote the names of the amino acid residues corresponding to each small molecule.

The protein structures of aspartame and eight core targets (APLNR, CYP2C19, EDNRA, KLK5, F2R, RAD51, AURKA, and TLR2) were systematically validated through molecular docking. The findings demonstrated a favorable binding affinity between aspartame and all eight target proteins (Table 2), with binding energies lower than −5.0 kcal/mol. It should be noted that these binding energy values represent computational estimates derived from the docking scoring function rather than experimentally determined thermodynamic binding free energies, as molecular docking algorithms employ simplified scoring functions that cannot fully capture the complexity of protein–ligand interactions in biological systems. Furthermore, visualization of the binding conformations (Figure 9A–H) revealed that all aspartame–protein complexes exhibited stable docking conformations, thereby providing a structural foundation for elucidating the mechanism of intermolecular interactions.

## 3. Discussion

In recent years, the widespread use of food additives has led to a series of food safety incidents, raising public concern regarding their safety. Among these additives, the health risks associated with the artificial sweetener aspartame, particularly its potential carcinogenicity, have been the subject of intense debate [16]. Reports indicate that exposure to aspartame is linked to the development of several cancers, including RCC, low-grade glioma, breast cancer, and prostate cancer, and that it may influence cancer incidence through multiple pathways [17]. Specifically, aspartame is metabolized by digestive enzymes to yield phenylalanine, aspartic acid, and methanol in humans. Methanol can subsequently be oxidized in the liver to formaldehyde, formic acid, and other metabolites [18]. Phenylalanine, as the primary metabolite, may contribute to cancer development through metabolic processes that generate ROS, thereby inducing oxidative stress and DNA damage. Additionally, it can promote tumor growth by enhancing cell proliferation and inhibiting apoptosis [19]. Formaldehyde, a by-product of metabolism, interacts with cellular components such as DNA and proteins, resulting in gene mutations and cellular damage. It also affects the signaling pathways involved in cell growth, differentiation, and apoptosis, which may further contribute to carcinogenesis [3]. Thus, exposure to aspartame is suggested to create initial conditions conducive to the development of certain cancers. Although current epidemiological studies indicate that aspartame exposure may elevate the risk of RCC, including KIRP, the precise molecular mechanism remains unclear [6]. Furthermore, the academic community presents varying conclusions regarding its carcinogenic effects. Therefore, a comprehensive elucidation of the molecular mechanisms by which aspartame contributes to the development of KIRP is crucial for resolving the ongoing controversy surrounding its safety and potential carcinogenicity.

To address this research gap, this study systematically investigated the potential molecular mechanisms of aspartame in KIRP utilizing network toxicology and molecular docking methods. Initially, we integrated multiple online databases to identify potential targets, yielding a total of 61 intersecting target genes associated with KIRP linked to aspartame. The potential biological functions of these target genes were further clarified through GO and KEGG pathway enrichment analyses. The results indicate that aspartame may promote remodeling of the KIRP tumor microenvironment and accelerate disease progression by regulating key processes such as extracellular matrix degradation, signaling pathway transduction, protein metabolism, and the immune microenvironment. Subsequently, unifactorial Cox regression analysis identified 23 genes significantly associated with patient prognosis. Moreover, multifactorial Cox regression analysis with stepwise regression revealed 8 core target genes (*APLNR*, *CYP2C19*, *EDNRA*, *KLK5*, *F2R*, *RAD51*, *AURKA*, and *TLR2*) involved in the mechanism linking aspartame to KIRP. A risk prediction model was constructed for these 8 genes by combining their regression coefficients. The rationality and clinical potential of the model were subsequently validated through SHAP analysis and survival analysis. SHAP analysis revealed that *EDNRA* (SHAP value = 0.664) served as the core driver gene of the model, elucidating the relationship between the expression of each gene and individual risk, as well as the heterogeneity of predictions. This analysis provided a mechanistic framework that supports clinical translation. Furthermore, survival analysis demonstrated that the model effectively differentiates the prognostic risk among KIRP patients, indicating that both the risk score and tumor stage (particularly stages III and IV) function as independent prognostic factors for survival. This finding offers potential biomarkers and a theoretical foundation for clinical prognostic assessment and therapeutic decision-making in KIRP. Additionally, the biological relevance of these findings was corroborated using molecular docking methods, which indicated a strong binding affinity between aspartame and the core gene product, further substantiating its potential oncogenic role in KIRP. In summary, our results suggest that the identified core genes may represent key molecular targets for the oncogenic effects of aspartame in KIRP, thereby providing a theoretical basis for future clinical research and treatment strategies.

The core genes identified in this study demonstrated multifaceted roles in aspartame-induced KIRP carcinoma. Among them, APLNR, a G protein-coupled receptor prevalent in human tissues, regulates various physiological processes, including angiogenesis, cardiovascular function, and metabolism, primarily through the Apelin/APLNR signaling pathway. Aberrant expression of APLNR may influence the tumor microenvironment and diminish the efficacy of cancer immunotherapies [20]. Tolkach et al. [21] revealed that APLNR expression is negatively correlated with tumor invasiveness in RCC patients. Furthermore, higher expression levels of APLNR were observed in high-grade, high-stage, and metastatic RCC, indicating its potential as an independent prognostic indicator for assessing patient survival. The molecular docking results from this study indicate that aspartame can bind stably to APLNR, suggesting its involvement in the mechanism of aspartame-induced KIRP by modulating the angiogenic and proliferative capabilities of KIRP cells. CYP2C19, a member of the CYP450 family, plays a crucial role in the metabolism of various exogenous and endogenous substances. The downregulation of CYP2C19 expression not only diminishes the liver and kidney’s ability to metabolize and detoxify but also results in the accumulation of harmful metabolites, which can lead to cellular damage and oxidative stress. Furthermore, this downregulation impacts the metabolism of arachidonic acid and related inflammatory mediators, thereby exacerbating local inflammation and promoting the progression of RCC [22,23]. AlRasheed et al. [24] demonstrated that a reduction in CYP450 activity in experimental rats, following four weeks of continuous exposure to varying doses of aspartame (175 or 1000 mg/kg/d), adversely affected the metabolism of drugs such as tyrosine kinase inhibitors (TKIs), resulting in decreased efficacy. The molecular docking results from this study indicate that aspartame can stably bind to CYP2C19, suggesting that it may influence the response of KIRP cells to carcinogens by disrupting the metabolic function of CYP2C19, thereby providing a potential molecular basis for the development of KIRP. EDNRA serves as a crucial receptor within the endothelin signaling pathway, which regulates vasoconstriction, cell survival, and inflammatory responses [25]. Furthermore, studies have demonstrated that in aggressive tumors such as bladder and gastric cancers, EDNRA expression is influenced by the upregulation of the STAT3 signaling pathway. This interaction establishes a positive feedback loop that enhances tumor cell proliferation, invasion, migration, epithelial–mesenchymal transition, and drug resistance [26]. Conversely, downregulating EDNRA expression can significantly inhibit tumor cell growth, migration, and apoptosis, suggesting its potential as an anti-tumor target [27]. Although direct evidence linking the regulation of EDNRA expression to KIRP therapy is lacking, its high expression and critical regulatory roles across various cancers warrant further investigation as a therapeutic target for KIRP. Additionally, the molecular docking results from this study indicate that aspartame binds stably to EDNRA, leading to the hypothesis that it may influence inflammatory responses and microangiogenesis in KIRP cells by modulating EDNRA function. This interaction presents a potential target for understanding the molecular oncogenesis associated with aspartame. KLK5, a serine protease within the human kinin-releasing kinase (KLK) gene family, plays a significant role in protein degradation and cellular signal transduction, and is closely associated with tumorigenesis, development, invasion, and metastasis [28,29]. Tailor et al. [30] observed that various KLK proteins exhibited cytoplasmic immunohistochemical expression in renal tubular epithelial cells. Furthermore, they reported that KIK gene expression was markedly altered across all three types of RCC, with the downregulation of the KLK5 gene being closely linked to the progression of KIRP. In conjunction with the molecular docking results from this study, aspartame demonstrated stable binding to KLK5, indicating that it may influence the invasive capacity of KIRP cells by modulating KLK5 activity, thereby presenting the potential to serve as an aspartame-associated biomarker for KIRP. F2R, also known as protease-activated receptor 1 (PAR1), is a G-protein-coupled receptor that plays a critical role in coagulation, inflammation, and cancer progression, including the migration, invasion, and proliferation of cancer cells [31]. Kaufmann et al. [32] demonstrated that PAR1 is significantly expressed in renal carcinoma cells from patients with RCC and is predominantly localized in cellular membranes and intracellular compartments. Bergmann et al. [33] further revealed that PAR1 mediates the tyrosine phosphorylation of the epidermal growth factor receptor (EGFR) in human renal cancer cells, thereby enhancing their migratory capacity. In conjunction with the molecular docking results presented in this study, aspartame is shown to bind stably to F2R. It is hypothesized that aspartame may influence the migratory behavior of renal cancer cells by modulating the PAR1/EGFR-related pathway, thereby providing a foundation for elucidating the molecular mechanisms underlying KIRP and for the development of potential therapeutic targets. RAD51, a critical protein involved in DNA homologous recombination repair, is vital for maintaining genomic stability and is frequently overexpressed in various cancers [34]. Liu et al. [35] reported that the expression of the SET structural domain monomethyltransferase was significantly upregulated, while RAD51 expression was notably downregulated, accompanied by an increase in lysine methylation levels in surgical samples of RCC, including KIRP, consistent with the characteristic features of RCC. In conjunction with the molecular docking results of the present study, aspartame demonstrates a stable binding affinity for RAD51. Although direct evidence linking aspartame to the regulation of RAD51 or SET structural domain monomethyltransferases is lacking, it is posited that aspartame may indirectly influence the DNA repair capacity and genomic stability of KIRP cells by modulating RAD51 function, potentially affecting the progression of KIRP. AURKA is a serine/threonine kinase that plays a critical role in the cell cycle, mitosis, and various other biological processes. It is closely linked to the development of several cancers, including RCC [36,37]. Wen et al. [38] demonstrated that AURKA expression is significantly upregulated in human RCC tissues and cells, correlating with poor prognosis. Furthermore, the knockdown of AURKA effectively inhibits the proliferation and migration of RCC cells, induces apoptosis, and causes cell cycle arrest in the G1/G2 phase. This process is accompanied by the downregulation of Bcl-2, CCNB1, CCND1, and CCNA1 expression. Mechanistic studies further revealed that AURKA influences RCC progression by regulating the transcription of CCNB1. Additionally, the results of molecular docking in this study indicate that aspartame can stably bind to AURKA, suggesting that it may impact KIRP progression by modulating AURKA’s kinase activity. This modulation could subsequently affect the division and proliferation of KIRP cells as well as the transcriptional activity of CCNB1; however, the underlying mechanisms require further experimental validation. TLR2, a crucial receptor in innate immunity, recognizes pathogens and initiates inflammatory responses, thereby playing a significant role in immune regulation and being closely associated with cancer development [39]. Ping et al. [40] found that TLR2 is significantly expressed across various tumor types, including KIRP. Wang et al. [41] further demonstrated that elevated TLR2 expression is present in renal cancer tissues and adjacent non-cancerous tissues of renal cancer patients, with high expression levels positively correlating with reduced patient survival rates. Combined with the molecular docking results of the present study, aspartame is shown to bind stably to TLR2. It is hypothesized that aspartame may accelerate the progression of KIRP by influencing cellular immune regulation and inflammatory responses, suggesting its potential as a biomarker and therapeutic target for the diagnosis and treatment of KIRP.

The synergistic effects of the aforementioned core target genes indicate a complex network that encompasses cell cycle abnormalities, inflammatory responses, metabolic dysregulation, vascular function, and tumorigenesis. This network offers a theoretical foundation and identifies potential targets for future mechanistic studies, targeted interventions, and novel drug development.

This study systematically investigated the potential targets and mechanisms of action associated with aspartame-induced KIRP utilizing network toxicology methods. This approach offers a novel perspective to address the existing research gap and elucidate the relationship between the two. Nonetheless, the study has several limitations: (1) Although internal validation using a training-validation split approach demonstrated the generalizability of the risk model, external validation using independent cohorts (such as datasets from the GEO database) was not performed due to the scarcity of KIRP-specific datasets with complete survival information in public repositories. Future multicenter studies should validate the model using external cohorts to further confirm its clinical applicability. (2) There is no standardized protocol for the acquisition and processing of raw target data, making target information vulnerable to variations arising from database sources, algorithmic modeling, and screening criteria, which may result in biased outcomes. (3) The binding affinity of aspartame to core target proteins primarily depends on actual biological activity, which has not been further validated through in vitro and in vivo experiments. (4) The molecular docking analysis employed a semi-flexible approach where the ligand was flexible, but the receptor backbone and sidechains remained rigid. Incorporating full receptor flexibility, particularly sidechain flexibility in the binding site, might yield different binding energies and binding poses. Additionally, only the top-ranked docking pose was selected for analysis, while alternative binding conformations from lower-ranked poses were not examined. Flexible docking approaches combined with analysis of multiple binding poses or molecular dynamics simulations would provide more comprehensive insights into the dynamic nature and conformational diversity of aspartame-protein interactions. (5) The network toxicology analysis relies predominantly on static data, failing to account for the temporal dynamics of aspartame exposure; for instance, short-term and long-term exposure may exert differing effects on target activation. (6) This study lacks experimental evidence to support a direct association between certain core targets and the potential mechanisms involved in the induction of KIRP by aspartame. Furthermore, the causality of the proposed mechanism chain requires additional verification. (7) The study does not elucidate the effects of varying exposure dosages on the activation of the targets or pathways. The effective dosage of aspartame during actual exposure may be influenced by individual metabolic differences and other factors, necessitating further experimental investigation to explore the dose–effect relationship. (8) The absence of data derived from real-world clinical cohorts may compromise the reliability of epidemiological correlation predictions.

Follow-up studies can further elucidate the specific roles of core genes in the development of KIRP by employing cell and animal models with core genes either knocked out or knocked in, alongside functional experiments. Additionally, experiments involving multi-dose gradient exposure and time series can be designed to quantify the dynamic changes in core gene expression, pathway activation, and disease phenotype, thereby revealing the timing and quantitative patterns of effects. Concurrently, further standardized multicenter clinical cohort studies can be conducted to unify nadir criteria and detection indices, validating the predictive performance of the model using multicenter data or external cohorts. This approach will also facilitate the integration of data to analyze the relationships between core genes and the clinical features of KIRP, ultimately enhancing the clinical translational value. In conclusion, this study establishes a preliminary framework for elucidating the potential mechanisms underlying aspartame-induced KIRP; however, the results require further validation through experimental studies, thereby laying the groundwork for the development of prevention and treatment strategies.

## 4. Materials and Methods

### 4.1. Collection and Analysis of KIRP-Related Data in the TCGA Database

RNA-seq data and clinical information for KIRP were retrieved from the TCGA database (https://portal.gdc.cancer.gov/; last accessed: 13 September 2025) and subsequently downloaded. The expression matrices and clinical data were organized and merged by sample ID to ensure correspondence. As all transcriptomic data were obtained from a single source (TCGA), which applies standardized data processing pipelines, no additional batch effect correction was performed. Differential expression analysis was conducted in R 4.5.1 (R Foundation for Statistical Computing, Vienna, Austria) utilizing the “DESeq2” (version 1.50.2) and “edgeR” (version 4.8.0) packages. Differentially expressed genes were identified using the criteria *p* < 0.05 and |log2(FC)| > 1. Visualization of these genes was accomplished through heatmaps and volcano plots, generated with the “pheatmap” (version 1.0.13) and “ggplot2” (version 4.0.1) packages in R, respectively.

### 4.2. Target Screening for Aspartame and KIRP

The PubChem database (https://pubchem.ncbi.nlm.nih.gov/; last accessed: 29 July 2025) was queried using the keyword “Aspartame” to retrieve its chemical structure and SMILES formula (COC(=O)C@HNC(=O)C@HN). This molecular information served as input data for the ChEMBL (https://www.ebi.ac.uk/chembl/; last accessed: 29 July 2025), SwissTargetPrediction (http://www.swisstargetprediction.ch/; last accessed: 29 July 2025), and SEA (https://sea.bkslab.org/; last accessed: 29 July 2025) databases to predict potential targets, with a focus on human (Homo sapiens) species. For ChEMBL, all predicted targets for aspartame in humans were retained without additional filtering criteria. In the SwissTargetPrediction and SEA databases, only target genes with a predicted probability or score greater than 0.05 were retained for aspartame. Additionally, KIRP-related target genes were gathered from the GeneCards (https://www.genecards.org/; last accessed: 13 September 2025), OMIM (https://omim.org/; last accessed: 13 September 2025), and CTD (https://ctdbase.org/; last accessed: 13 September 2025) databases using the term “kidney renal papillary cell carcinoma” to collect KIRP-related target information. For GeneCards, only protein-coding genes with a Relevance score > 5 were retained. For OMIM, all disease-associated genes (Approved Symbols) were extracted. For CTD, only targets with an Inference score > 5 were retained. Finally, the gene datasets were merged, deduplicated, and standardized using the UniProt database (https://www.uniprot.org/uniprotkb; last accessed: 13 September 2025) to obtain the target gene sets for aspartame and KIRP. Specifically, gene identifiers from different sources (gene symbols, Ensembl IDs, etc.) were uniformly mapped to UniProt accessions using the UniProt ID mapping tool (https://www.uniprot.org/id-mapping; last accessed: 13 September 2025), with species restricted to Homo sapiens. Duplicate entries and ambiguous mappings were removed to generate standardized target gene sets.

### 4.3. Screening of Target Genes for the Intersection of Aspartame and KIRP

The differentially expressed genes obtained from TCGA, along with the target genes of aspartame and related targets of KIRP, were integrated and analyzed for intersections. All gene identifiers were standardized to official gene symbols prior to intersection analysis to ensure consistency across datasets. The intersected target genes potentially implicated in the pathogenesis of KIRP due to aspartame were identified. A Venn diagram was created and visualized using the “ggvenn” (version 0.1.19) package in R.

### 4.4. GO and KEGG Enrichment Analysis of Intersecting Target Genes

We employed the “clusterProfiler” package (version 4.18.2) for Gene Ontology (GO) functional annotation and Kyoto Encyclopedia of Genes and Genomes (KEGG) pathway enrichment analysis. The “enrichplot” package (version 1.30.4) facilitated result visualization, while the “Org.Hs.eg.db” package (version 2.4.6) served as the human gene annotation database. The GO analyses encompassed biological processes (BP), cellular components (CC), and molecular functions (MF), which elucidated the potential functions of the target genes across various biological levels. In contrast, the KEGG analyses aimed to identify the principal metabolic pathways and signaling mechanisms associated with the intersected target genes. A significance threshold of *p* < 0.05 was established to ensure the reliability of the enrichment results.

### 4.5. PPI Network Construction and Analysis

Protein–protein interaction (PPI) analysis was conducted on the screened intersecting target genes utilizing the STRING database (https://string-db.org/; last accessed: 14 September 2025). The analysis was restricted to “Homo sapiens,” with a confidence score threshold established at ≥0.4, and free nodes were concealed to retain only biologically significant interactions within the network. Subsequently, the PPI network files were imported into Cytoscape v3.10.3 software (Cytoscape Consortium, La Jolla, CA, USA), organized by the degree value, which represents the number of interactions between nodes, and the PPI network was visualized and analyzed.

### 4.6. Cox Regression Analysis and Prognostic Risk Modeling

The “tidyverse” package (version 2.0.0) in R was initially employed to integrate gene expression data and survival information, including survival time and status, for the intersecting gene list and KIRP samples from the TCGA database, based on sample ID. Only samples from the tumor group were retained, resulting in the generation of merged result files. Subsequently, the “survival” (version 3.8.3) and “survminer” (version 0.5.1) packages were utilized to read the merged data, and a unifactorial Cox proportional hazards regression analysis was conducted for each intersected target gene. Genes significantly correlated with patient prognosis were identified (*p* < 0.05), and their expression data were extracted for the subsequent construction of risk models. Finally, a forest plot was created using the “forestplot” package (version 3.1.7) to visualize the hazard ratios (HR) and 95% confidence intervals (CIs) of the genes, illustrating the distribution of risk genes (HR > 1) and protective genes (HR < 1).

Prior to multifactorial Cox regression analysis, multicollinearity among the 23 prognostically significant genes was assessed using the variance inflation factor (VIF). A linear regression model was constructed with survival time as the dependent variable and gene expression values as independent variables. VIF values were calculated using the “car” package (version 3.1.3) in R, with VIF ≤ 5 indicating acceptable collinearity levels and VIF > 10 indicating severe multicollinearity [42].

In constructing the prognostic risk model, significant genes identified through unifactorial Cox regression analysis were incorporated into a multifactorial Cox regression model. Subsequently, two-way stepwise regression, guided by the Akaike Information Criterion (AIC), was conducted using the step function in R to identify the core prognostic-related genes included in the model along with their regression coefficients (*βi*). Following model construction, the proportional hazards assumption was evaluated using Schoenfeld residuals. The cox.zph function from the “survival” package (version 3.8.3) was employed to calculate the chi-square statistic and corresponding *p*-value for each covariate and for the global model. A *p*-value > 0.05 indicated that the proportional hazards assumption was satisfied, confirming that the hazard ratios remained constant over the follow-up period. The risk score formula was derived from the multifactorial Cox regression results:

$$risk\;score=\sum_{i=1}^{n}\beta i\times xi$$

where *n* represents the number of genes in the model, *xi* denotes the expression level of the *i*th gene in the corresponding sample, and *βi* indicates the associated regression coefficient. The surv_cutpoint function from the “survminer” package (version 0.5.1) was employed to calculate the cutoff point of the survival curve that exhibited the greatest difference, thereby determining the optimal cutoff value. Consequently, samples were categorized into high and low-risk groups for subsequent survival analysis. Additionally, the “pheatmap” package (version 1.0.13) was utilized to arrange the samples according to their risk scores, from low to high. Risk score curves, survival distribution maps, and heatmaps of risk gene expression were generated to visualize the impact of the risk model on patient survival status and gene expression characteristics.

### 4.7. Risk Model Interpretability Analysis (SHAP)

Utilizing the multigene risk prediction model developed in Section 4.6, this study employed the SHAP algorithm to quantify the contribution of each gene to model predictions and enhance interpretability. Initially, the R language “glmnet” package (version 4.1.0) was utilized to screen features and standardize gene data, ensuring the comparability of subsequent analyses. This was combined with the “survival” package (version 3.8.3) to construct the Cox regression survival model. Following this, the “kernelshap” (version 0.9.1) and “shapviz” packages (version 0.10.3) were employed to calculate the SHAP values for genes associated with the risk scores of the Cox model, allowing for a quantitative assessment of both positive and negative contributions of genes to the risk scores and their relative importance. Finally, the “ggplot2” package (version 4.0.1) was used to generate bar charts, summary plots, and force plots for individual samples, thereby visualizing the contribution of each feature to the model output.

### 4.8. Risk Model Survival Analysis and Effectiveness Assessment

Based on the risk score file obtained in Section 4.6, the survival object and Kaplan–Meier (K-M) survival curve were constructed utilizing the Surv and Survfit functions from the “survival” package (version 3.8.3) in R. The survival curves were visualized through the ggsurvplot function of the “survminer” package (version 0.5.1), and the differences in survival among various risk groups were assessed using the log-rank test. Additionally, the ggsurvplot function was employed to illustrate the survival differences between high- and low-risk groups, with statistical significance determined at *p* < 0.05 to evaluate the impact of risk grouping on patient prognosis. Furthermore, time-dependent receiver operating characteristic (ROC) curve analysis was conducted using the “timeROC” package (version 0.4) in R. The survival prediction efficacy of the model was assessed at the 1-, 3-, and 5-year intervals, with the area under the curve (AUC) calculated for each corresponding time point. A larger AUC value indicates a higher prediction accuracy of the model at that specific time point. Additionally, we incorporated risk scores alongside patients’ clinicopathologic characteristics (e.g., age, gender, stage) in multifactorial Cox regression analyses to evaluate the independent prognostic significance of risk scores on the overall survival of patients with KIRP and to identify other independent risk factors. Finally, based on the analysis results, forest plots were generated to illustrate the impact of each risk factor (age, gender, stage, and risk group) on the overall survival of KIRP patients.

### 4.9. Internal Validation

To assess the generalizability of the 8-gene risk model, internal validation was performed using a training-validation split approach. The 278 KIRP samples were randomly divided into a training set (*n* = 194, 70%) and a validation set (*n* = 84, 30%) using a fixed random seed (seed = 123) in R to ensure reproducibility. The risk score for each sample was calculated using the regression coefficients (*βi*) derived from the original multifactorial Cox regression model: risk score = Σ(*βi* × *xi*), where xi represents the expression level of the ith gene. Patients were stratified into high- and low-risk groups based on the median risk score of the training set. Kaplan–Meier survival curves were generated for both training and validation sets using the “survminer” package (version 0.5.1), and survival differences between risk groups were compared using the log-rank test. Time-dependent ROC curves were constructed to evaluate the predictive accuracy of the model at 1, 3, and 5 years using the “timeROC” package (version 0.4), with corresponding AUC values calculated.

### 4.10. Molecular Docking Validation

Molecular docking was employed to further validate the interaction between aspartame and core target gene proteins. Initially, the three-dimensional (3D) structure of aspartame, downloaded in SDF format in 2.2, served as the ligand. The 3D structural data of the core target gene proteins were subsequently acquired from the RCSB PDB (https://www.rcsb.org/pages/about-us/index; last accessed: 15 September 2025) database to function as the receptor. The protein structure was then pre-processed using PyMol 2.6.0 software (Schrödinger, LLC, New York, NY, USA), which involved the removal of water molecules and hydrogenation to optimize its conformation. Prior to docking, the dimensions and position of the docking cassette were established using the center coordinates of the active pocket as a reference, in conjunction with the ligand molecular size and the volume of the active pocket. Following the preparation of the protein and ligand, molecular docking was conducted using AutoDock Vina 1.1.2 software (The Scripps Research Institute, La Jolla, CA, USA) with a semi-flexible docking approach, where the ligand was treated as flexible while the receptor backbone and sidechains remained rigid. This approach, rather than fully rigid body docking, allows ligand conformational flexibility while maintaining computational efficiency. It was selected as it provides a reasonable balance between computational cost and accuracy for preliminary screening of protein–ligand interactions in network toxicology studies, and is consistent with established practices in similar computational analyses. For each protein–ligand pair, AutoDock Vina generated multiple docking poses ranked by binding energy. The top-ranked pose with the lowest binding energy was selected as the representative binding conformation for subsequent analysis, which is a standard practice in network toxicology and network pharmacology studies for preliminary target screening. A binding energy threshold of ≤−5.0 kcal/mol was used to indicate favorable binding stability between the receptor and ligand. Finally, the resulting docking data were imported into PyMol for visualization, emphasizing the binding sites of the ligand on the target protein and the key types of interactions.

## 5. Conclusions

This study systematically evaluated the potential association between the artificial sweetener aspartame and the pathogenesis of KIRP using a network toxicology analytical framework, alongside bioinformatics analysis and molecular docking techniques. The results indicate that aspartame may contribute to the pathogenesis of KIRP by targeting specific genes and pathways. Furthermore, molecular docking analyses demonstrated that aspartame can form stable bindings with the proteins encoded by the eight core target genes identified. This finding establishes a theoretical foundation for further exploration of the molecular mechanisms underlying aspartame-induced KIRP. Future research should focus on the relationship between the duration and dosage of aspartame exposure and the risk of KIRP development. Additionally, it is essential to validate the predictive performance of the model through in vivo and in vitro functional experiments, as well as clinical cohort studies. Such efforts will clarify the specific mechanisms by which the core target genes are involved in aspartame-associated KIRP, potentially identifying targets for interventions aimed at mitigating the adverse effects of aspartame on renal health, as well as its use as a food additive.

## Figures and Tables

**Table 1:** Risk model genes and regression coefficients.

Gene Name	Coeff
*APLNR*	−0.285
*CYP2C19*	0.245
*EDNRA*	0.576
*KLK5*	0.149
*F2R*	0.375
*RAD51*	0.403
*AURKA*	0.378
*TLR2*	−0.188

Note: Positive coefficients indicate risk factors (higher expression associated with increased mortality risk), while negative coefficients indicate protective factors (higher expression associated with reduced mortality risk).

**Table 2:** Binding energies of ligand and receptor.

Ligand	Receptor	Binding Energy (kcal/mol)
Aspartame	APLNR	−6.8
Aspartame	CYP2C19	−8.0
Aspartame	EDNRA	−6.7
Aspartame	KLK5	−5.7
Aspartame	F2R	−6.2
Aspartame	RAD51	−7.0
Aspartame	AURKA	−6.9
Aspartame	TLR2	−7.4

**Figure A1:**
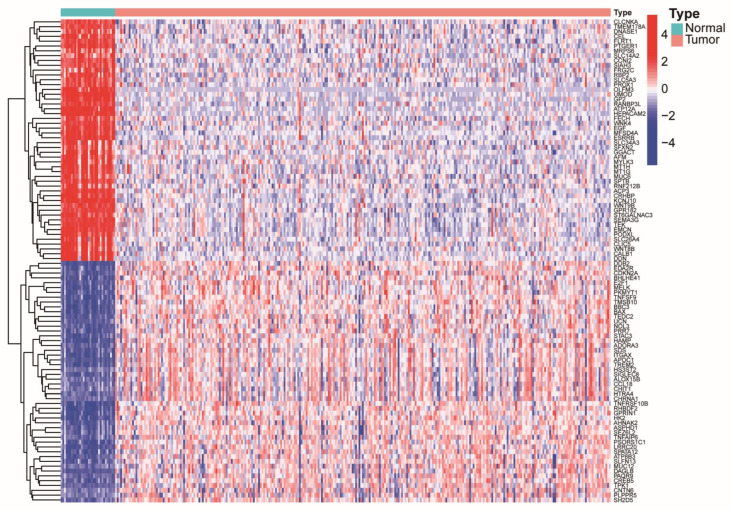
Complete heatmap of differentially expressed genes.

## Data Availability

The original contributions of this study are included in the article. For further inquiries, contact the corresponding author.

## Supplementary Materials

The following supporting information can be downloaded at: https://www.mdpi.com/article/10.3390/ijms27010077/s1.

## Appendix A. Complete Heatmap of Differentially Expressed Genes

Figure A1 displays expression patterns of the top 50 most significantly upregulated and top 50 downregulated genes across 291 tumor samples and 32 normal samples. Expression values were z-score normalized, with red indicating high expression and blue indicating low expression. Hierarchical clustering was applied to both rows (genes) and columns (samples). The top annotation bar indicates sample type: Normal (cyan) and Tumor (pink).
